# Morphine Exacerbates Experimental Colitis-Induced Depression of Nesting in Mice

**DOI:** 10.3389/fpain.2021.738499

**Published:** 2021-12-13

**Authors:** Stanley M. Cheatham, Karan H. Muchhala, Eda Koseli, Joanna C. Jacob, Essie Komla, S. Stevens Negus, Hamid I. Akbarali

**Affiliations:** Department of Pharmacology and Toxicology, Virginia Commonwealth University, Richmond, VA, United States

**Keywords:** opioids, depression, inflammation, analgesia, tolerance, colitis, inflammatory bowel disease

## Abstract

Opioids and non-steroidal anti-inflammatory drugs (NSAIDs) are excellent analgesics, but recent clinical evidence suggests that these drugs might worsen disease severity in Crohn's disease patients, limiting their clinical utility for treating Inflammatory Bowel Disease (IBD). One indicator of change in well-being from conditions such as IBD is behavioral depression and disruption to activities of daily living. Preclinical measures of behavioral depression can provide an indicator of changes in quality of life and subsequent modification by candidate analgesics. In mice, nesting is an adaptive unconditioned behavior that is susceptible to disruption by noxious stimuli, and some types of pain related nesting depression are responsive to opioid and NSAID analgesics. Here we show that a 2, 4, 6-trinitrobenzene sulfonic acid (TNBS) model of experimental colitis depresses nesting behavior in mice, and we evaluated effects of morphine, an opioid, and ketoprofen, a NSAID, on TNBS-induced nesting depression. In Swiss Webster mice, TNBS significantly reduced nesting that peaked on Day 3 and recovered in a time-dependent manner with complete recovery by Day 7. In the absence of colonic inflammation, daily treatment with morphine (1–10 mg/kg) did not decrease nesting except at 10mg/kg/day. However, in TNBS-treated mice 3.2 mg/kg/day morphine significantly exacerbated TNBS-induced nesting depression and delayed recovery. While 3.2 mg/kg/day morphine alone did not alter locomotor activity and TNBS-induced depression of locomotion recovered, the combination of TNBS and 3.2 mg/kg/day morphine significantly attenuated locomotion and prevented recovery. Daily treatment with 3.2 or 10 mg/kg ketoprofen in TNBS-treated mice did not prevent depression of nesting. These data suggest that opioid analgesics but not NSAIDS worsen colonic inflammation-induced behavioral depression. Furthermore, these findings highlight the importance of evaluating analgesic effects in models of colonic inflammation induced depression of behavior.

## Introduction

Nest building is an adaptive “activity of daily life” (ADL) in mice (1, 2). Mice, including those used in research laboratories, innately build nests to protect themselves from predators, conserve heat, and shelter from environmental stressors (2). Several studies have reported that mice under distress exhibit reduced nesting behavior (3, 4). Thus, impairment of nesting can serve as a valuable dependent measurement to assess health and welfare of mice (4). Moreover, nesting in mice can also be depressed by some models of experimental pain, such as intraperitoneal injection of dilute lactic acid (IP acid) as a model of acute visceral pain. IP acid-induced nesting depression is alleviated by both mu opioid receptor (MOR) agonist analgesics and non-steroidal anti-inflammatory drugs (NSAIDs) (5–7).

Inflammatory bowel diseases (IBD), which comprises Crohn's disease and ulcerative colitis, is characterized by chronic recurrent inflammation and ulceration of the bowels (8). It is widely believed that in IBD the homeostatic interaction between the mucosal immune system and commensal gut flora is perturbed such that an exaggerated immune response to bacterial antigens produces gastrointestinal inflammation and disrupts the gut epithelial barrier (9). This results in extraintestinal inflammation that contributes to inflammation-induced pain (10–12). Unfortunately, strategies to alleviate pain in IBD patients are limited because MOR agonist analgesics such as morphine have been linked to poor disease outcomes and increased mortality (8, 13–17). Analysis of the Crohn's Therapy, Resource, Evaluation and Assessment Tool (TREAT) registry of over 6,000 patients, showed that narcotic use was associated with increased risk of infection (hazard ratio = 1.98) and mortality (18). NSAIDs such as cyclooxygenase (COX) inhibitors constitute a non-opioid class of analgesics commonly used to treat many types of inflammatory pain, but especially non-selective inhibitors of the COX_1_ and COX_2_ isoenzymes may also adversely affect IBD (15, 19, 20). It is however unclear whether poor quality of life and impaired activity of daily living in IBD patients using opioid analgesics or NSAIDs are due to direct effects of these drugs on behavior or to interactions between the drugs and the underlying IBD.

One model of experimental colonic inflammation in mice is intracolonic treatment with 2, 4, 6-trinitrobenzene sulfonic acid (TNBS). Assessment of pain-related behaviors in the TNBS treated mice, includes a hypersensitive withdrawal responses from tactile stimuli applied to the abdomen as a type of pain-stimulated behavior in mice; however, in contrast to the clinical contraindication of MOR agonists for IBD treatment, morphine reduced this tactile hypersensitivity (21). Given this evidence for poor translational validity of drug effects on pain-stimulated behavior in the TNBS mouse model, we tested whether TNBS treatment would also produce behavioral depression. In this study, we determined whether TNBS-induced colonic inflammation impairs nest building and locomotor activity in mice, and we determined the effectiveness of morphine and the NSAID/COX_1/2_ inhibitor ketoprofen to mitigate any colitis-induced nesting impairment. The study proceeded in three steps: 1) we evaluated nesting and locomotor activity for 7 days after treatment with 2, 4, 6-trinitrobenzene sulfonic acid (TNBS) 2) we determined the effect of daily morphine treatment on nesting and locomotor activity in the absence and presence of colonic inflammation, and 3) we similarly determined the effect of daily ketoprofen treatment on nesting in the presence of colonic inflammation.

## Methods

### Animals

Male Swiss Webster mice *(Envigo, Indianapolis, IN)* ~8 weeks old (24–39 g) were housed in cohorts of five in temperature- and humidity-controlled animal care quarters under a 12-hour light/ dark cycle (lights on from 7 AM−7 PM). Each cage contained one 5 × 5 cm^2^ “nestlet” composed of pressed cotton, and mice had ad libitum access to food and water. All animal procedures were reviewed and approved by the Institutional Animal Care and Use Committee at Virginia Commonwealth University (VCU IACUC).

### Experimental Model of Colitis

Twenty-four hours before initiation of testing, animals were lightly anesthetized with isoflurane (1–2 min), and a stainless-steel bulb-tipped gavage needle coated in petroleum jelly was inserted into the distal colon at a distance of up to 3 cm from the rectum to deliver 100 μL of 2.5% 2, 4, 6-trinitrobenzenesulfonic acid (TNBS) or vehicle (1:1 ETOH: saline), as reported previously (22). After administration, the mice were held elevated by the tail for 30s to prevent displacement of the injected solution. Mice were then group-housed in clean cages and allowed to recover.

### Nesting Procedure

**Figure 1:**
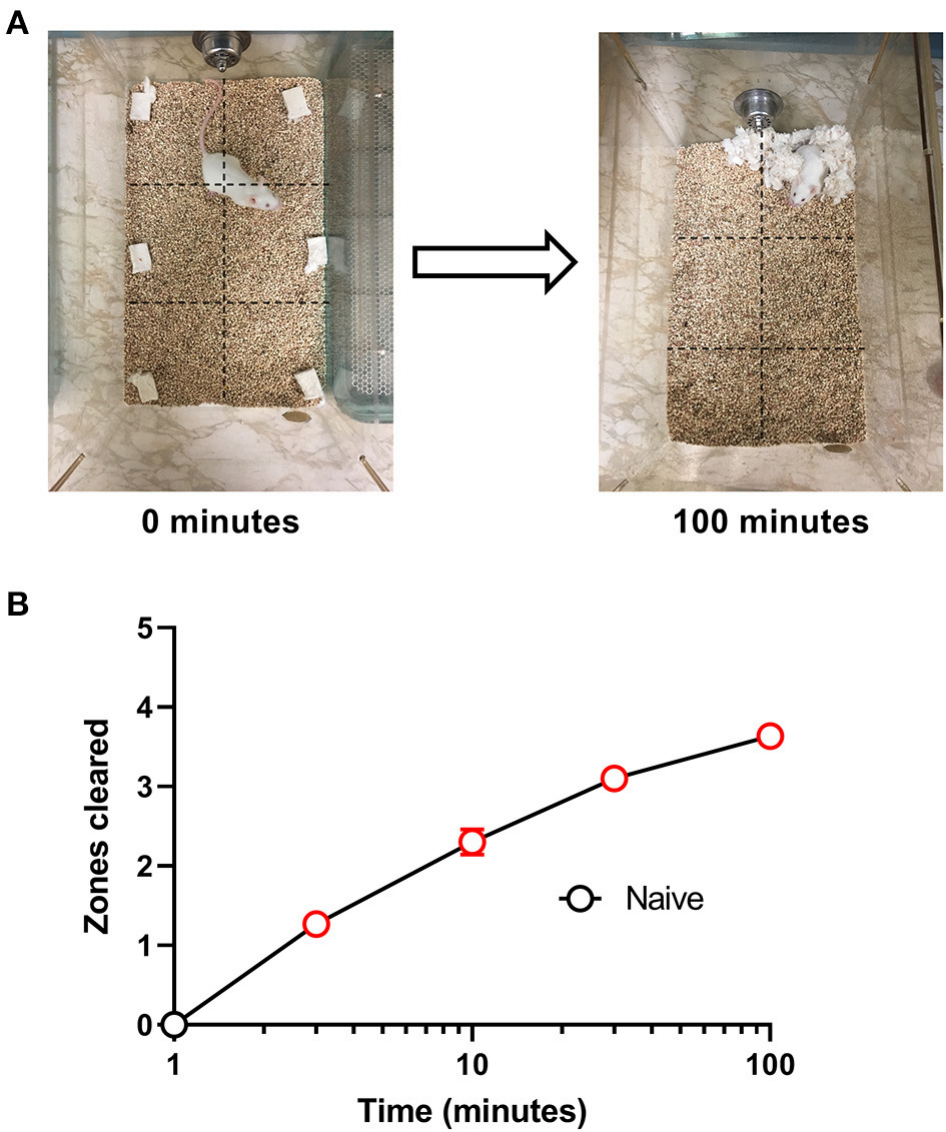
Nesting in naïve mice. **(A)** Representative images of nesting behavior at time 0 min and at the end of the experiment at 100 min. Nestlets were placed within the cage in six equally sized zones indicated by the dotted grid lines, and the number of zones cleared of their nestlet was determined at 1, 3, 10, 30, and 100 min. The maximum number of zones that can be cleared is 5. **(B)** Nestlet consolidation can be quantified as a time-dependent increase in zones cleared. Red points indicate significance (*P* < 0.05) vs. “time = 1 min” by repeated measures one-way ANOVA with Dunnett's test. Data are mean ± SEM. *N* = 30 for every time point.

All experiments were conducted during the light cycle. On test days, mice were transferred from the housing room and acclimated to the windowless testing room for 10 min. Each mouse was then removed from its home cage and placed individually in a test cage with corn cob bedding and free access to food and water for 100-min nesting sessions as described previously (6). Prior to the start of each session, mice were weighed and a 5 × 5 cm^2^ nestlet was divided into six equal pieces that were distributed across a grid of six equally sized “zones” across the cage floor (Figure 1). Under control conditions, mice consolidate the nestlet pieces as the first step in nest building, and this nestlet consolidation was quantified using a measure of “zones cleared” as the primary dependent variable. Thus, at the start of a session, each zone contains a nestlet piece, and the number of zones cleared was “0.” Over time, mice clear nestlets out of some zones and consolidate them into others, and the maximum number of zones cleared is 5 (i.e., nestlet pieces cleared out of 5 zones and consolidated into the one remaining zone). The number of zones cleared was recorded at times of 1, 3, 10, 30, and 100 min.

Nesting was determined daily for 7 days in each cohort of mice starting one day after administration of TNBS or vehicle. Mice were administered morphine, ketoprofen, or saline injections 30 min before the start of 100 min nesting sessions on each day for 7 days. Body weights were evaluated daily for 7 days and mice were returned to their home cages and then to the housing room after each test session. Loss in body weight is one of the major indices of disease activity index with TNBS treatment.

High doses of morphine (10 mg/kg) in TNBS-treated mice caused a rapid decline in body weight to the maximum permissible 20 percent weight loss and resulted in mortality in some animals. Hence, in compliance with our IACUC protocols we used only those mice that survived for 7 days without exceeding the 20% weight loss limit (Table 1).

**Table 1:** Nesting was determined in mice randomly assigned to the following groups.

**Treatment**	***N* (completed study)**
Naive	30
Vehicle	6
TNBS	7
Saline (s.c.)	6
Morphine (1.0 mg/kg s.c.)	6
Morphine (3.2 mg/kg s.c.)	7
Morphine (10 mg/kg s.c.)	5
TNBS + Saline	8
TNBS + Morphine (1.0 mg/kg s.c.)	6
TNBS + Morphine (3.2 mg/kg s.c.)	6
TNBS + Saline	8
TNBS + ketoprofen (3.2 mg/kg s.c.)	7
TNBS + ketoprofen (10 mg/kg s.c.)	5
Placebo	9
75 mg morphine pellet	12

### Locomotor Activity

Spontaneous locomotor activity was measured for 30 min in individual boxes 27 × 27 × 20.3 cm with 16 infrared (I/R) beams housed in enclosed, sound attenuating activity chambers (ENV-510) (Med Associates, St. Albans, VT) that record ambulatory counts via infrared photo beam breaks. The primary dependent variable was distance traveled (in cm). Distance traveled was recorded in 5-min blocks for 30 min. Three cohorts of Swiss Webster mice (*n* = 6/cohort) were used in this assay, a) Morphine (3.2 mg/kg s.c. daily), b) TNBS + saline, and c) TNBS + morphine (3.2 mg/kg s.c. daily). Locomotor activity was determined at baseline for all groups prior to TNBS instillation and/or morphine daily injections. Locomotor activity was measured on day 1, 3, 5, and 7 following TNBS instillation. Mice were acclimatized to the recording room for 1 h prior to start of experiments. Locomotor assays were carried out 30 min following morphine injection.

### Test Drugs

TNBS (Sigma-Aldrich,St Louis, MO) was dissolved in 1:1 100% ethanol: 0.9%saline for intracolonic administration 24 h before testing was to begin. Morphine sulfate (National Institute on Drug Abuse Drug Supply Program, Bethesda, MD) and a commercially available 100 mg/ml ketoprofen solution (L-arginine,70 mg, citric acid, benzyl alcohol, 0.025 g; Zoetis, Spain) were diluted in saline vehicle. Saline, Morphine or Ketoprofen were administered subcutaneously 30 min before mice were placed into testing cages. The test drugs and combination are as follows: Saline, 1.0 mg/kg morphine, 3.2 mg/kg morphine, 10 mg/kg morphine, TNBS, TNBS vehicle, TNBS+ Saline, TNBS + 1.0 mg/kg morphine, TNBS + 3.2 mg/kg morphine, TNBS + 3.2 mg/kg Ketoprofen, TNBS + 10 mg/kg ketoprofen, placebo pelleted and 75 mg morphine pelleted.

For chronic morphine administration, morphine (75 mg) or placebo pellets were implanted subcutaneously on the dorsum. The pellets were obtained from the National Institute of Drug Abuse (NIDA). Surgical implantation of the pellets was done under anesthesia (2.5% isoflurane). The hair on the base of the neck was shaved and the skin thoroughly cleansed with 10% providing iodine (General Medical Corp. Walnut, CA) and then rinsed with 70% ethanol. A 1 cm horizontal incision was made at the cleansed area, and the pellet was inserted into the subcutaneous space. The site was closed by stapling the skin with Clay Adams Bran, MikRon Autoclip 9 mm Wound clips (Beckton Dickson, Franklin Lakes, NJ). The mice were allowed to recover in their home cages and tested for nesting the following day.

### Data Analysis

Nesting: Nesting behavior was acquired as “zones cleared” across time at 1, 3, 10, 30, and 100 min for each respective day over the 7-day period. Data are also presented as the area under curve (AUC) of the zones cleared from 0–30 minutes determined for each day and plotted across the 7 days. These data were evaluated by two-way repeated measures ANOVA with treatment and time as the two independent variables. Multiple comparisons between treatment groups were made using the Bonferroni's *post hoc* test and within-treatment comparisons with a single control group were made using the Dunnett's *post-hoc* test, as indicated in the figure legends.

Body weight: Changes in body weight were measured as percent change from baseline over the course of 7 days, and these data were also analyzed by two-way ANOVA as described above, with day after intracolonic injection as a within-subjects variable and treatment as a between-subjects variable.

Locomotor activity: Total distance traveled in 30 min was determined for each mouse as described above. These data were evaluated by fitting a mixed model using Restricted Maximum Likelihood (REML) instead of a repeated-measures two-way ANOVA due to the presence of missing values in the TNBS + 3.2 mg/kg/day morphine group on Day 7. Two mice treated with TNBS + morphine died on day 7. Treatment and time were the two independent variables. The Bonferroni's *post hoc* test was used for multiple comparisons between treatment groups and the Dunnett's *post hoc* test was used for a within-treatment comparison across time with baseline activity.

The criterion for statistical significance for all analyses was *P* < 0.05. *Post-hoc* analysis of the ANOVA was performed only for significant main effects or significant interactions. All data analysis was performed with GraphPad Prism (version 9.0.1). Data are presented as mean ± SEM.

## Results

### Nesting Behavior in Naïve Mice

We initially tested the time course for the nesting behavior in Swiss Webster mice. Figure 1 shows that naive mice consolidated nestlets from six different zones into one corner of the cage in a time-dependent manner [F (4, 116) = 133.4, *P* < 0.001]. The mean ± SEM zones cleared after 100 min was 3.63 ± 0.15.

### TNBS Depressed Nesting Behavior

**Figure 2:**
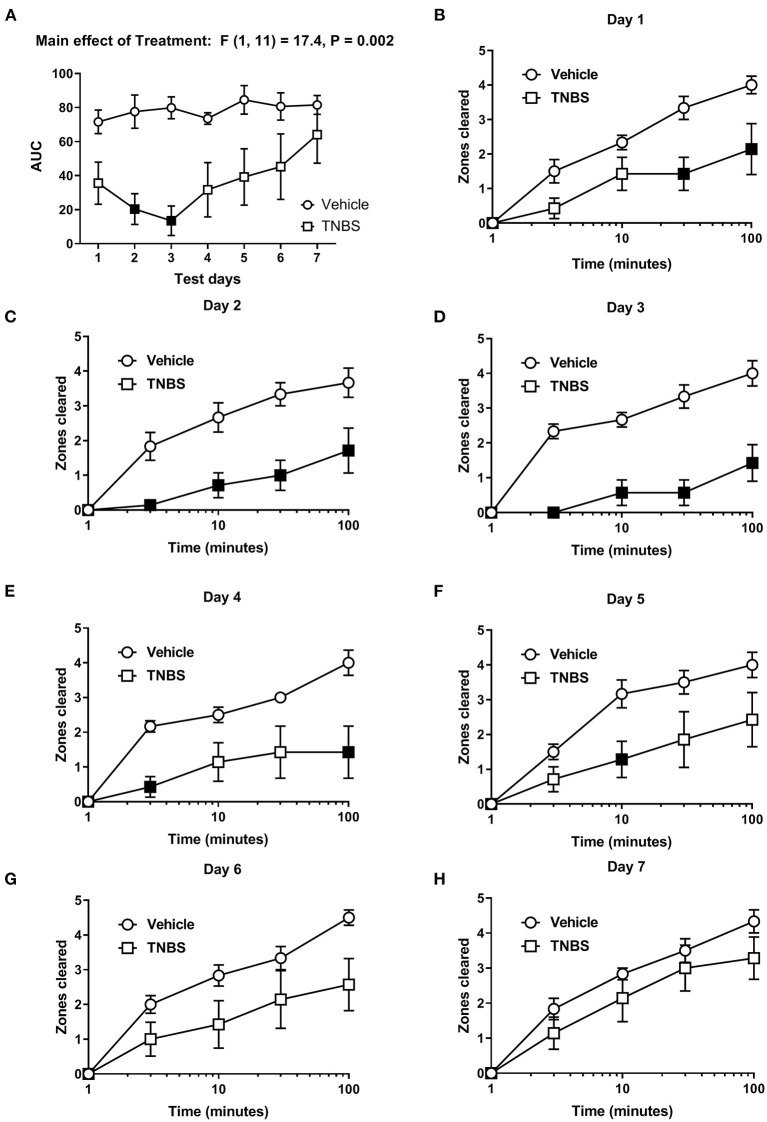
Effect of TNBS on nesting behavior. **(A)** The area under the curve (AUC) of nesting scores recorded daily in mice treated with vehicle or TNBS. **(B–H)** show zones cleared at 3, 10, 30, and 100 min in mice treated with vehicle or TNBS on Days 1–7, respectively. TNBS treatment transiently depressed nesting that peaked on Day 3 but was not apparent on Day 7. Filled points represent statistical significance (*P* < 0.05) vs. vehicle as indicated by a significant treatment effect in the two-way repeated measures ANOVA with Bonferroni's test. Data are mean ± SEM. Vehicle (*N* = 6/day); TNBS (*N* = 7/day).

In order to test the effect of colonic inflammation on nesting, the number of zones cleared was determined daily for 7 days following TNBS administration. To evaluate the overall effects of TNBS on the nesting behavior across the days, we determined the area under the curve (AUC) at each individual day. Figure 2 shows the day-wise AUC of nesting in vehicle- and TNBS-treated mice (A) and the time course of nesting in vehicle- and TNBS-treated mice on each day (B-H). Relative to the vehicle treatment, TNBS produced a transient decrease in nesting that peaked on Day 3 (Figure 2A) and returned by Day 7. A two-way ANOVA comparing the day-wise AUC of nesting in vehicle- and TNBS-treated mice indicated a main effect of treatment [F (1, 11) = 17.4, *P* = 0.002], but not a significant time x treatment interaction [F (6, 66) = 1.1, *P* = 0.35].

For nesting assessed on individual days, we detected a significant time x treatment interaction on Day 1 [F (4, 44) = 4.36, *P* = 0.005], Day 2 [F (4, 44) = 5.3, *P* = 0.001], Day 3 [F (4, 44) = 9.2, *P* < 0.001], Day 4 [F (4, 44) = 4.9, *P* = 0.002] and Day 5 [F (4, 44) = 2.9, *P* = 0.03], thus indicating a significant inhibitory effect of TNBS treatment vs. vehicle on the time course of nesting on Days 1–5 (Figures 2B-F). Conversely, two-way ANOVA did not indicate a significant time × treatment interaction on Day 6 [F (4, 44) = 2.5, P 0.055] (Figure 2G) or Day 7 [F (4, 44) = 0.8, *P* = 0.55] (Figure 2H), nor did it indicate a main effect of TNBS treatment on Day 6 [F (1, 11) = 3.4, *P* = 0.092] (Figure 2G) or Day 7 [F (1, 11) = 1.4, *P* = 0.27] (Figure 2H). These data suggest that TNBS depressed nesting that tended to recover over time.

**Figure 3:**
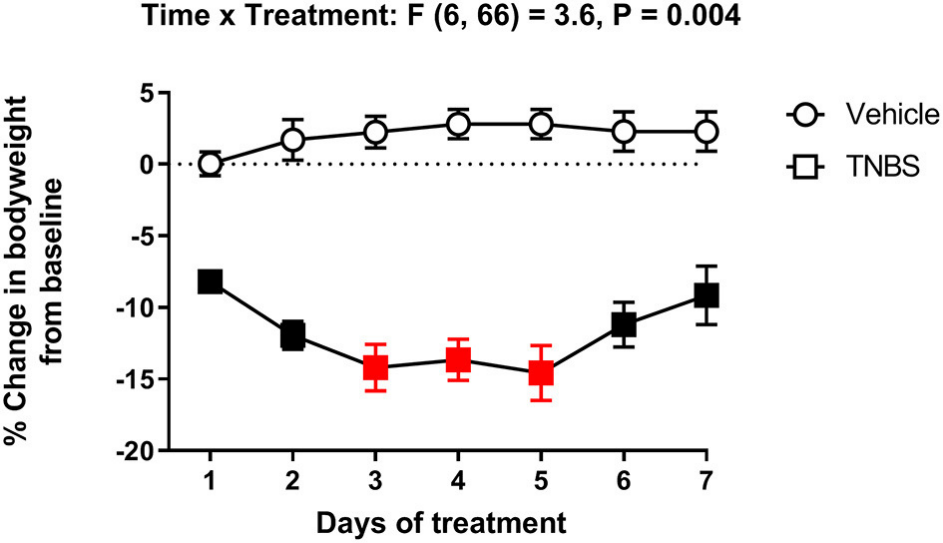
Effect of TNBS treatment on body weight. Percent change in body weight from baseline of mice treated with vehicle or TNBS. TNBS significantly reduced body weight that peaked on Day 3. Recovery of the lost body weight was observed starting Day 6. Filled points represent statistical significance (*P* < 0.05) vs. vehicle as indicated by a significant time x treatment interaction in the two-way repeated measures ANOVA with Bonferroni's test. Data are mean ± SEM. Vehicle (*N* = 6/day); TNBS (*N* = 7/day). Red points indicate significance (*P* < 0.05) vs. day one by repeated measures two-way ANOVA with Dunnett's test.

Figure 3 represents the percent change in body weight from baseline in mice treated with vehicle or TNBS. In concurrence with depression of nesting (Figure 2), TNBS treatment caused a progressive reduction of body weight that tended to peak on Day 3. Body weight of TNBS-treated mice recovered to Day 1 levels by Day 7, but were significantly reduced compared to that of vehicle controls. Two-way ANOVA of the percent change in body weight from baseline in mice treated with vehicle or TNBS indicated a significant main effect of TNBS treatment [F (1, 11) = 129.4, *P* < 0.001] and a significant time x treatment interaction [F (6, 66) = 3.6, *P* = 0.004].

### Effect of Repeated Morphine Treatment on Nesting

**Figure 4:**
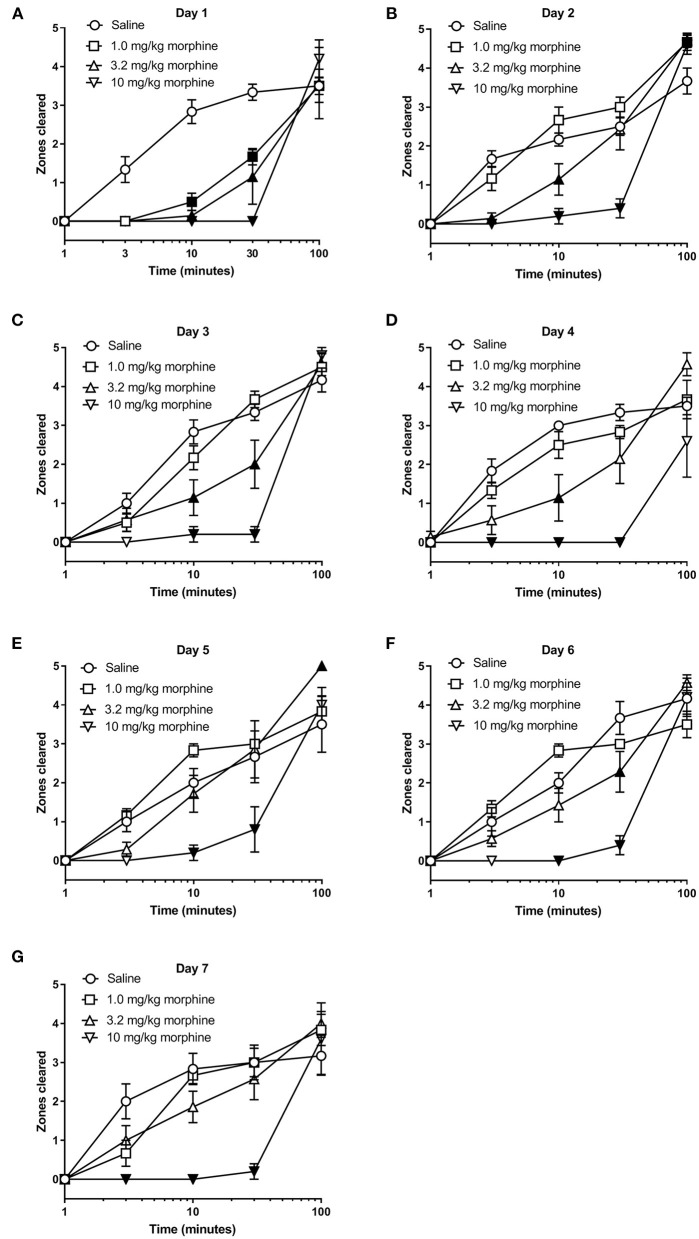
Effect of repeated morphine treatment on nesting behavior. **(A–G)** show zones cleared at 1, 3, 10, 30, and 100 min in mice treated daily with saline or morphine (1.0, 3.2 or 10 mg/kg/day) on Days 1–7, respectively. Nesting scores were recorded 30 min after saline or morphine treatment. 1.0 or 3.2 mg//kg morphine-induced depression of nesting peaked on Day 1 and was not apparent on Day 5. Alternatively, 10 mg/kg morphine significantly depressed nesting on all test days. Filled points represent statistical significance (*P* < 0.05) vs. saline as indicated by a significant treatment x time interaction in the two-way repeated measures ANOVA with Bonferroni's test. Sample sizes for the experimental groups are as follows: Saline (6/day), 1.0 mg/kg morphine (6/day), 3.2 mg/kg morphine (7/day) and 10 mg/kg morphine (5/day).

**Figure 5:**
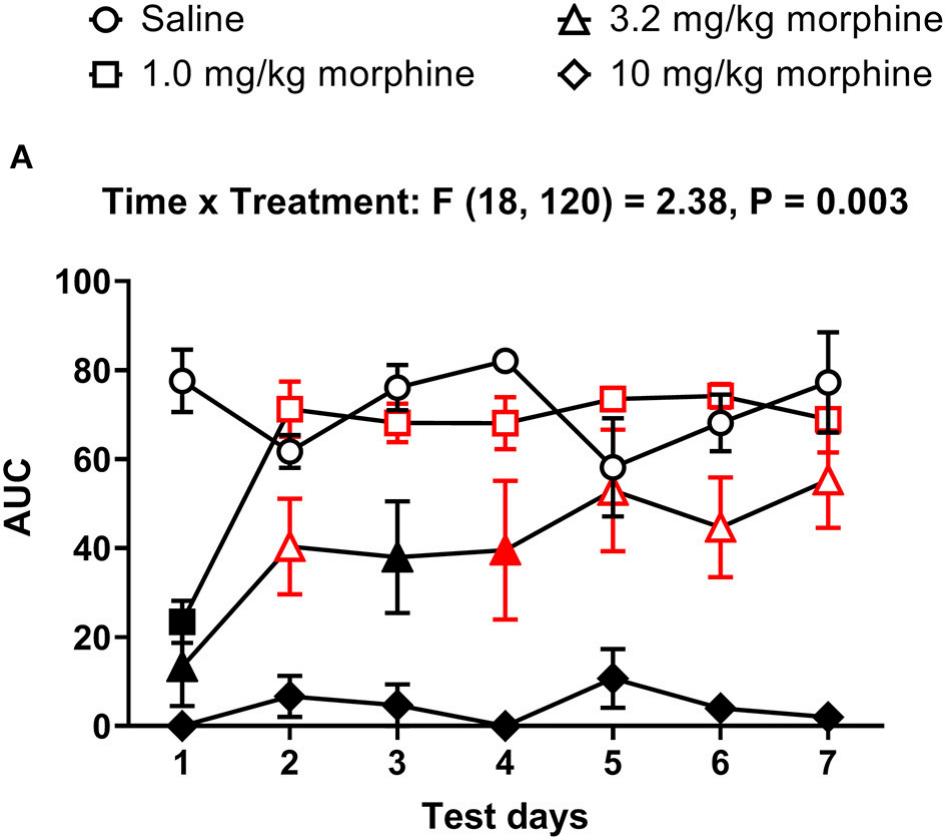
Time course of repeated morphine treatment on nesting behavior. **(A)** The area under the curve (AUC) of nesting scores recorded daily in mice treated with saline, 1.0, 3.2 or 10 mg/kg/day morphine s.c. Red points indicate significance (*P* < 0.05) vs. day one indicated by a significant time x treatment interaction by repeated measures two-way ANOVA with Dunnett's test. Filled points indicate significance vs. saline by Bonferroni's *post-hoc* analysis.

We next determined whether morphine treatment alone affected nesting behavior. Figures 4, 5 show the time-course of nesting on individual days and the day-wise AUC of nesting, respectively, in mice treated daily with saline, 1.0, 3.2 or 10 mg/kg morphine. On Day 1, all doses of morphine (1.0–10 mg/kg) depressed nesting compared to saline up to 30 min but recovered at 100 min (Figure 4A). While daily administration of 3.2 mg/kg morphine continued to depress nesting compared to saline up to Day 4 (Figures 4A–D), the number of zones cleared improved over time and returned to saline levels over the course of 7 days. On the other hand, daily administration of 10 mg/kg morphine depressed nesting on all test days (Figures 4A–G). Two-way ANOVA comparing zones cleared over 100 min in mice treated with saline, 1.0, 3.2 or 10 mg/kg/day morphine on each day indicated a significant time x treatment interaction for all test days (Day 1 [F (12, 80) = 4.8, *P* < 0.001], Day 2 [F (12, 80) = 8.0, *P* < 0.001], Day 3 [F (12, 80) = 8.2, *P* < 0.001], Day 4 [F (12, 80) = 6.0, *P* < 0.001], Day 5 [F (12, 80) = 3.7, *P* < 0.001], Day 6 [F (12, 80) = 7.8, *P* < 0.001] and Day 7 [F (12, 80) = 5.7, *P* < 0.001]).

In all treatment groups, nesting was depressed by morphine for up to 30 min but reverted to saline control values by 100 min, indicating recovery from morphine's effects (Figure 4). We therefore determined the AUC for each dose of morphine up to 30 min. The AUC data indicated that administration of 1.0 or 3.2 mg/kg/day morphine produced a significant decrease in nesting on Day 1 that recovered to saline control values over the days and was no longer apparent by Day 4; however, daily administration of 10 mg/kg morphine depressed nesting on all test days (Figure 5). Thus, two-way ANOVA analysis of the AUC of nesting in saline- or morphine-treated mice indicated significant main effects of treatment [F (3, 20) = 30.3, *P* < 0.001] and time [F (6, 120) = 4.1, *P* < 0.001], and a significant time x treatment interaction [F (18, 120) = 2.38, *P* = 0.003].

**Figure 6:**
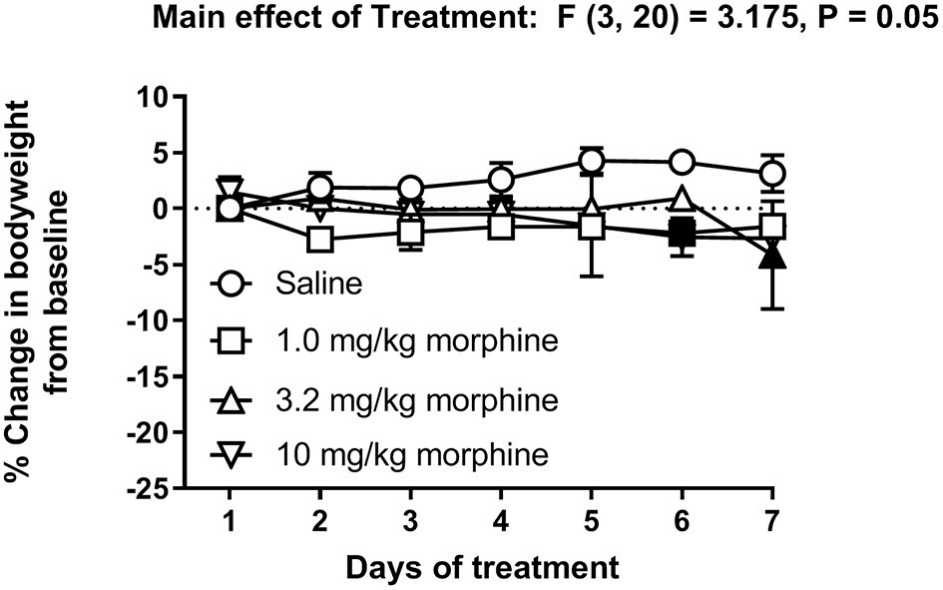
Effect of repeated morphine treatment on body weight. Percent change in body weight from baseline of mice treated repeatedly with saline, 1.0 mg/kg, 3.2 mg/kg or 10 mg/kg morphine s.c. Data are mean ± SEM and analyzed by two-way repeated measures ANOVA. Filled points indicate statistical significance (*P* < 0.05) vs. saline indicated by a main effect of treatment by repeated measures two-way ANOVA with Bonferroni's test. Saline (6/day), 1.0 mg/kg morphine (6/day), 3.2 mg/kg morphine (7/day) and 10 mg/kg morphine (5/day).

Figure 6 shows the percent change in body weight of mice treated daily with saline or morphine (1.0, 3.2 or 10 mg/kg). Morphine-treated mice had reduced body weights compared to saline-treated animals [Treatment effect: F (3, 20) = 3.2, *P* = 0.05], specifically on Days 6 and 7. However, two-way ANOVA comparing the percent change in body weight of saline or morphine treated mice did not detect a significant time x treatment interaction [F (18, 120) = 1.3, *P* = 0.21].

**Figure 7:**
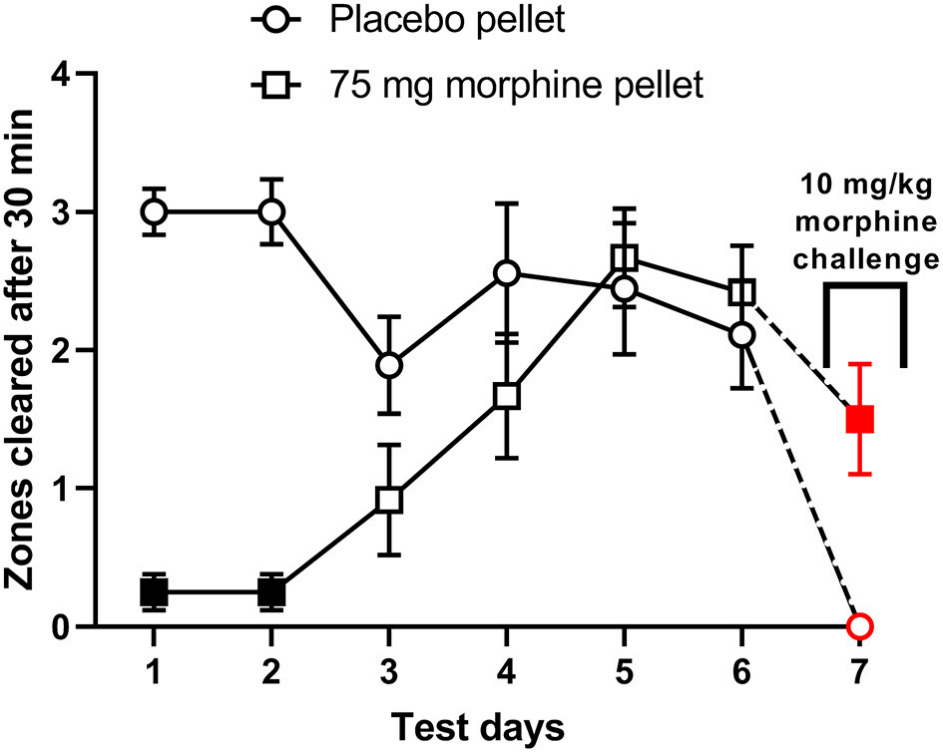
The effect of chronic morphine treatment on nesting behavior. Zones cleared from days 1–7 at the 30 min test mark in placebo (*n* = 9) and 75 mg morphine pelleted (*n* = 12) mice. On day 7 all mice received a “challenge” dose of 10 mg/kg morphine 30 min before the test period. Red points indicate significance (*P* < 0.05) vs. day 6 by repeated measures two-way ANOVA with Bonferroni's test. Filled points indicate significance vs. placebo indicated by a significant time x treatment interaction by repeated measures two-way ANOVA with Bonferroni's test.

The ability of morphine to suppress nesting behavior was further determined in mice implanted with placebo or morphine pellet (75 mg). Figure 7 shows that compared to placebo controls, the daily nesting behavior was significantly depressed in the morphine-pelleted mice on the first two days, but gradually recovered over the course of 6 days [Time × treatment: F (5, 95) = 9.7, *P* < 0.001]. On the 7th day, a 10 mg/kg challenge dose further depressed nesting in both morphine and placebo pelleted mice. However, the magnitude of depression of nesting caused by the 10 mg/kg challenge dose was significantly lower in the morphine-pelleted mice compared to placebo controls, indicating the development of tolerance.

### Morphine Exacerbated TNBS-Induced Depression of Nesting and Locomotion

**Figure 8:**
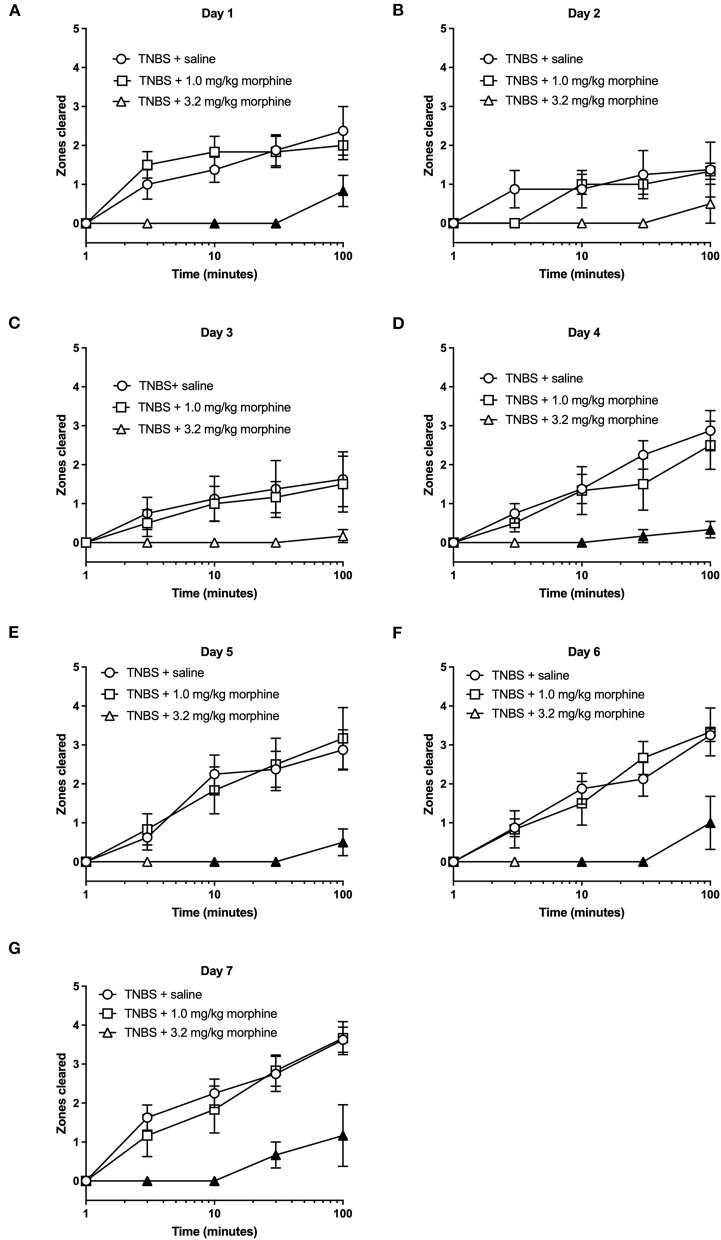
The effect of repeated morphine treatment on TNBS-induced depression of nesting. **(A–G)** show zones cleared at 1, 3, 10, 30, and 100 min in mice treated daily with saline or morphine (1.0, 3.2 or 10 mg/kg/day) on Days 1–7, respectively. Nesting scores were recorded 30 min after saline or morphine treatment. Compared to saline, 3.2, but not 1.0 mg//kg/day morphine significantly exacerbated TNBS-induced depression of nesting and prevented recovery of nesting behavior. Filled points represent statistical significance (*P* < 0.05) vs. saline as indicated by a significant treatment x time interaction in the two-way repeated measures ANOVA with Bonferroni's test. Data are mean ± SEM. Sample sizes for the experimental groups are as follows: TNBS + saline (*N* = 8/day); TNBS + 1.0 mg/kg morphine (*N* = 6/day); TNBS + 3.2 mg/kg morphine (*N* = 6/day).

We next tested whether repeated daily morphine treatment would affect TNBS-induced depression of nesting. In these set of experiments, we tested morphine at 1.0 and 3.2 mg/kg/day doses as 10 mg/kg induced mortality. Further, at the lower doses depression of nesting was transient when administered in the absence of colonic inflammation. Analysis of the time course of nesting in TNBS-treated mice injected with saline or morphine on individual days showed that 1 mg/kg morphine was not different than TNBS treated with saline across the 7 days. On the other hand, 3.2 mg/kg/day morphine further depressed nesting on all days with significant difference from Day 4 onwards (Figure 8). Two-way ANOVA indicated a significant morphine dose x time interaction on Day 1 [F (8, 68) = 2.7, *P* = 0.012], Day 4 [F (8, 68) = 4.3, *P* < 0.001], Day 5 [F (8, 68) = 4.7, *P* < 0.001], Day 6 [F (8, 68) = 4.3, *P* < 0.001] and Day 7 [F (8, 68) = 3.5, *P* = 0.002], but not on Day 2 [F (8, 68) = 1.7, *P* = 0.12] or Day 3 [F (8, 68) = 1.4, *P* = 0.2]. A main effect of morphine treatment was also not detected on Day 2 [F (2, 17) = 1.5, *P* = 0.25] or Day 3 [F (2, 17) = 1.7, *P* = 0.21]. This likely results from an already substantial depression by TNBS on Day 2 and Day 3.

**Figure 9:**
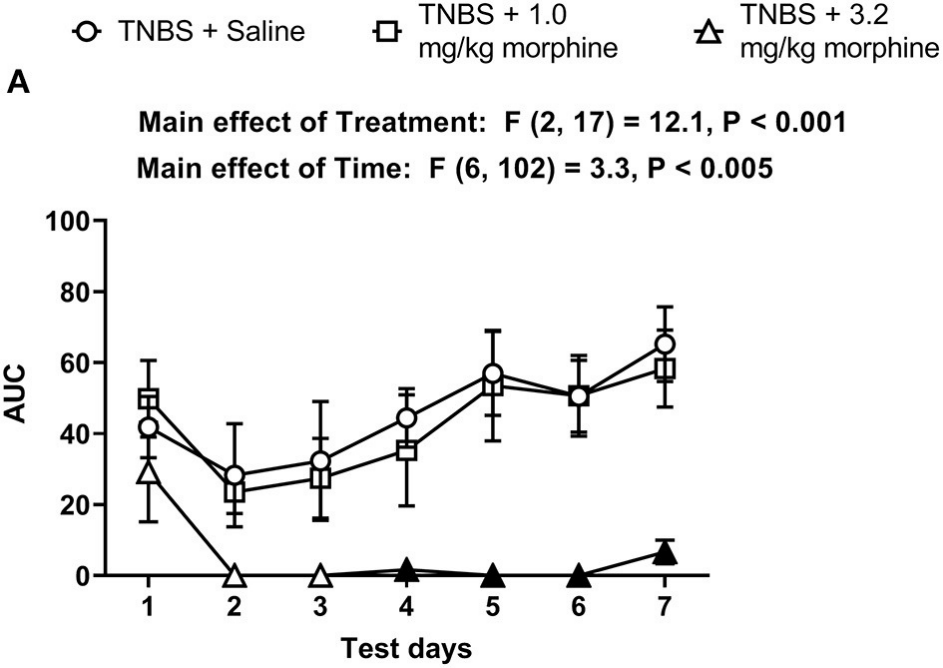
Time dependent effect of repeated morphine treatment on TNBS-induced depression of nesting. **(A)** The area under the curve (AUC) of nesting scores recorded daily in mice treated with TNBS and saline, 1.0 or 3.2 mg/kg/day morphine s.c. Filled points represent statistical significance (*P* < 0.05) vs. saline indicated by a main effect of treatment by repeated measures two-way ANOVA with Bonferroni's test. Data are mean ± SEM. Sample sizes for the experimental groups are as follows: TNBS + saline (*N* = 8/day); TNBS + 1.0 mg/kg morphine (*N* = 6/day); TNBS + 3.2 mg/kg morphine (*N* = 6/day).

The AUC for TNBS plus morphine across time shows that, 3.2 mg/kg/day morphine significantly exacerbated TNBS-induced nesting depression. This effect persisted up to 7 days (Figure 9). Two-way ANOVA analysis detected significant main effects of treatment [F (2, 17) = 12.1, *P* < 0.001] and time [F (6, 102) = 3.3, *P* = 0.005] but not a significant time x treatment interaction [F (12, 102) = 0.9, *P* = 0.51]. Consequently, these findings suggest that morphine dose-dependently exacerbates TNBS-induced depression of nesting and prevents recovery from nesting depression.

**Figure 10:**
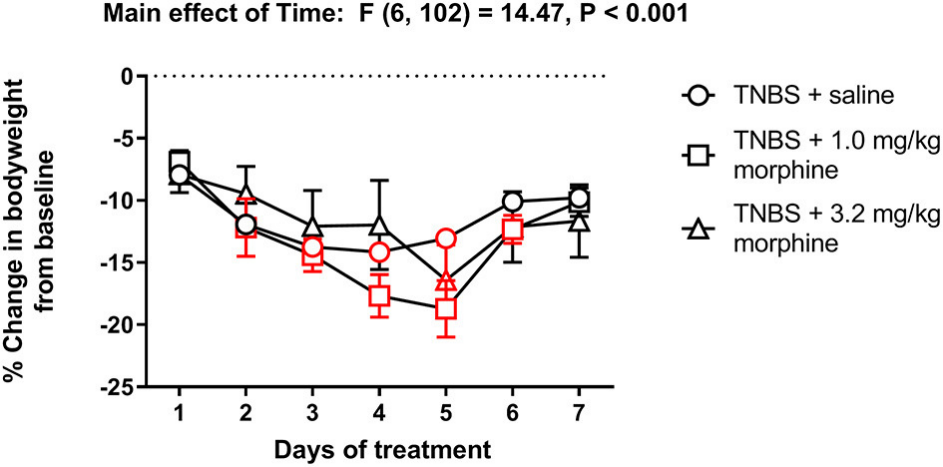
Effect of repeated morphine administration on the body weight of TNBS-treated mice. Percent change in body weight from baseline of TNBS-treated mice injected with saline, 1.0 or 3.2 mg/kg/day morphine s.c. Morphine did not exacerbate weight loss in TNBS-treated mice. Data are mean ± SEM and analyzed by two-way repeated measures ANOVA. TNBS + saline (*N* = 8/day); TNBS + 1.0 mg/kg morphine (*N* = 6/day); TNBS + 3.2 mg/kg morphine (*N* = 6/day). Red points indicate significance (*P* < 0.05) vs. day one by repeated measures two-way ANOVA with Dunnett's test.

Figure 10 shows the change in body weight of TNBS-treated mice injected with saline, 1.0 or 3.2 mg/kg/day morphine. Repeated treatment with morphine did not alter the trajectory of weight loss in TNBS-treated mice [Treatment effect: F (2, 17) = 0.2, *P* = 0.82]. All treatments caused a transient decrease in body weight that peaked on Day 4 in saline controls and Day 5 in morphine-treated animals, followed by recovery toward Day 1 levels on Day 7 [Time effect: F (6, 102) = 14.5, *P* < 0.001]. Also, no significant time x morphine treatment interaction was detected [F (12, 102) = 1.7, *P* = 0.07].

**Figure 11:**
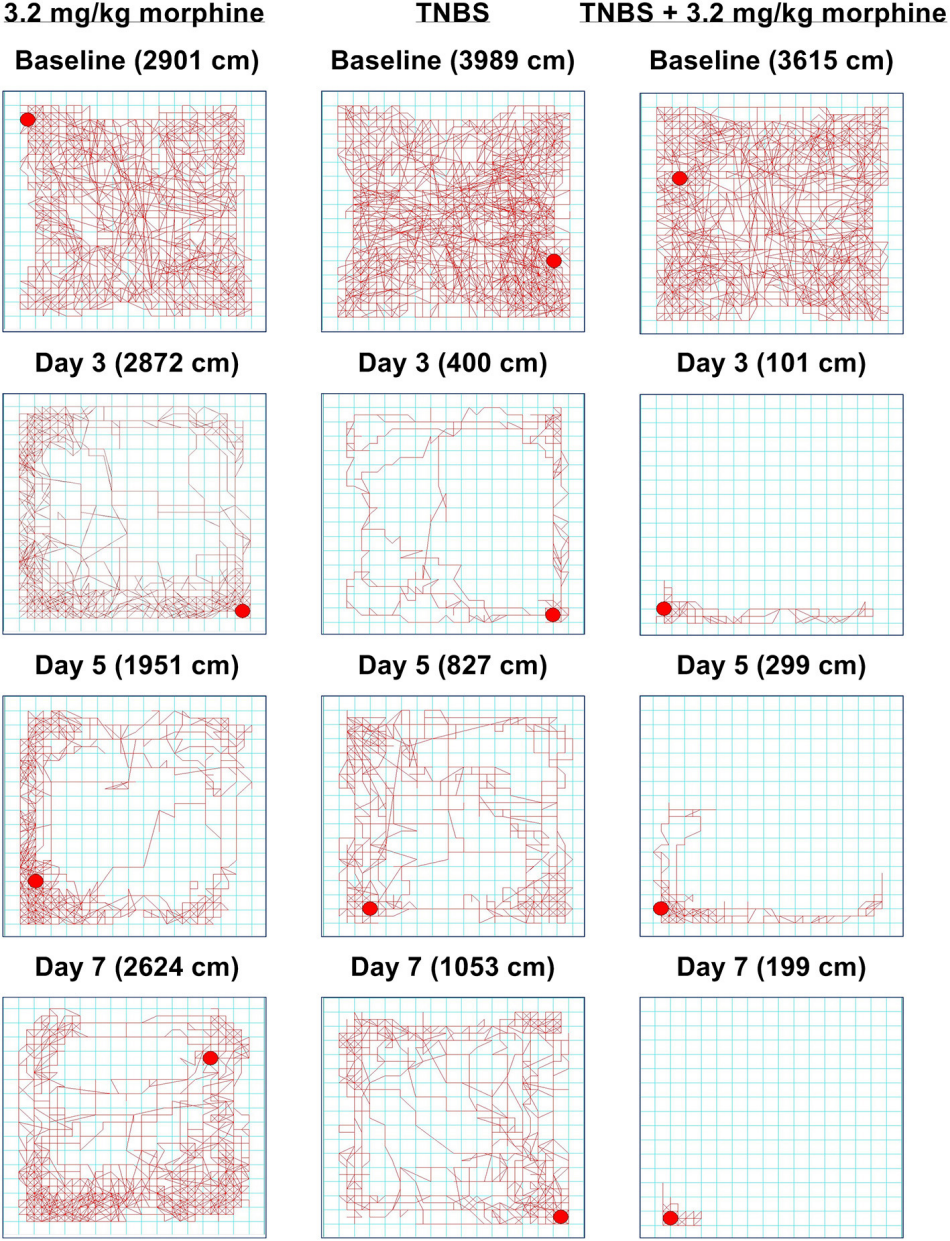
Time-dependent effect of morphine, TNBS and their combination on spontaneous locomotor activity. Locomotor activity in mice treated with (left) 3.2 mg/kg/day morphine, (center) TNBS and (right) TNBS + 3.2 mg/kg/day morphine at baseline, Day 3, Day 5, and Day 7. Distance traveled on each day is indicated in parenthesis. 3.2 mg/kg/day morphine does not disrupt locomotion, but the mouse appears to move along the perimeter, indicating thigmotaxis. Locomotor activity in TNBS-treated mouse is significantly attenuated, but appears to recover on Day 7. In the TNBS-treated mouse, daily morphine treatment exacerbates locomotor activity and delays recovery.

**Figure 12:**
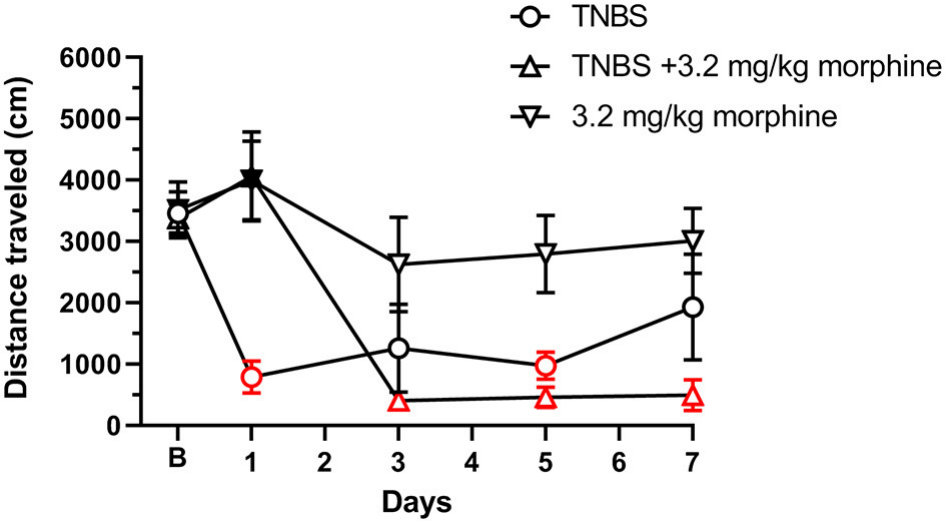
Daily administration of 3.2 mg/kg morphine exacerbates TNBS-induced depression of locomotor activity. Distance traveled (in cm) at 30 min recorded at baseline (denoted by “B”) and every alternate day for 7 days in mice treated with TNBS and/or 3.2 mg/kg/day morphine. Locomotor activity was measured 30 min after morphine injection. TNBS, but not morphine alone, significantly reduced locomotor activity that recovered to baseline on Day 7. Morphine on Day 1 only prevented the TNBS-induced depression of locomotor activity, however, repeated morphine treatment exacerbated TNBS-induced decrease in locomotion and prevented recovery to baseline. Data are mean ± SEM. TNBS (*N* = 6/day); 3.2 mg/kg morphine (*N* = 6/day); TNBS + 3.2 mg/kg morphine (*N* = 6 for Days 1, 3, and 5, and *N* = 4 for Day 7). *N* = 6/group for baseline. Red points indicate significance (*P* < 0.05) vs. B indicated by a significant time x treatment interaction by repeated measures two-way ANOVA with Dunnett's test. Filled points indicate significance vs. TNBS by Bonferroni's *post-hoc* analysis.

As with nesting behavior, TNBS also depressed locomotor activity from baseline with peak depression of locomotion observed on Day 3 and with recovery tending by Day 7 (Figures 11, 12). Morphine alone at 3.2 mg/kg did not significantly alter locomotor activity from baseline, although morphine-treated mice exhibited thigmotaxis (Figures 11, 12). In mice that received the combination of TNBS and morphine 3.2 mg/kg, there was no TNBS-induced depression of locomotor activity on Day 1, possibly due to the acute antinociceptive effect of morphine (Figure 12). However, locomotor activity was significantly depressed from baseline starting Day 3 and remained significantly depressed up to Day 7 i.e., similar to their effects on nesting behavior the combination of morphine and TNBS attenuated recovery from TNBS-induced depression of locomotion (Figures 11, 12). Thus, mixed-effects (REML) analysis of the data indicated a significant time x treatment interaction [F (8, 58) = 5.4. *P* < 0.001].

### Effect of Repeated Ketoprofen Treatment on TNBS-Induced Depression of Nesting

**Figure 13:**
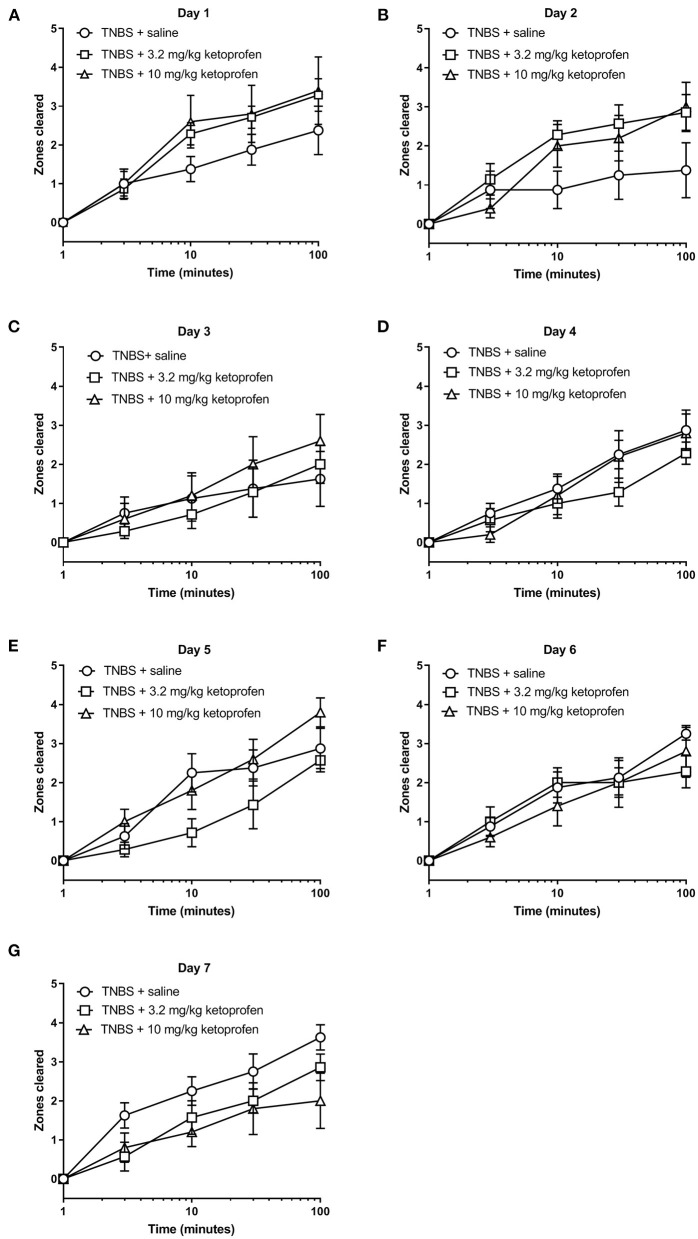
Effect of Ketoprofen on TNBS-induced nesting. **(A–G)** show zones cleared at 3, 10, 30, and 100 min in mice treated daily with saline or ketoprofen (3.2 or 10 mg/kg/day) on Days 1–7, respectively. Nesting scores were recorded 30 min after saline or ketoprofen treatment. Compared to saline, ketoprofen did not significantly alter nesting in TNBS-treated mice, except on Day 2, 3.2 mg/kg ketoprofen significantly mitigated TNBS-induced depressed nesting. Data are mean ± SEM. TNBS + saline (*N* = 8/day); TNBS + 3.2 mg/kg ketoprofen (*N* = 7/day); TNBS + 10 mg/kg ketoprofen (*N* = 5/day). Data are analyzed by two-way repeated measures ANOVA with Bonferroni's *post-test*.

**Figure 14:**
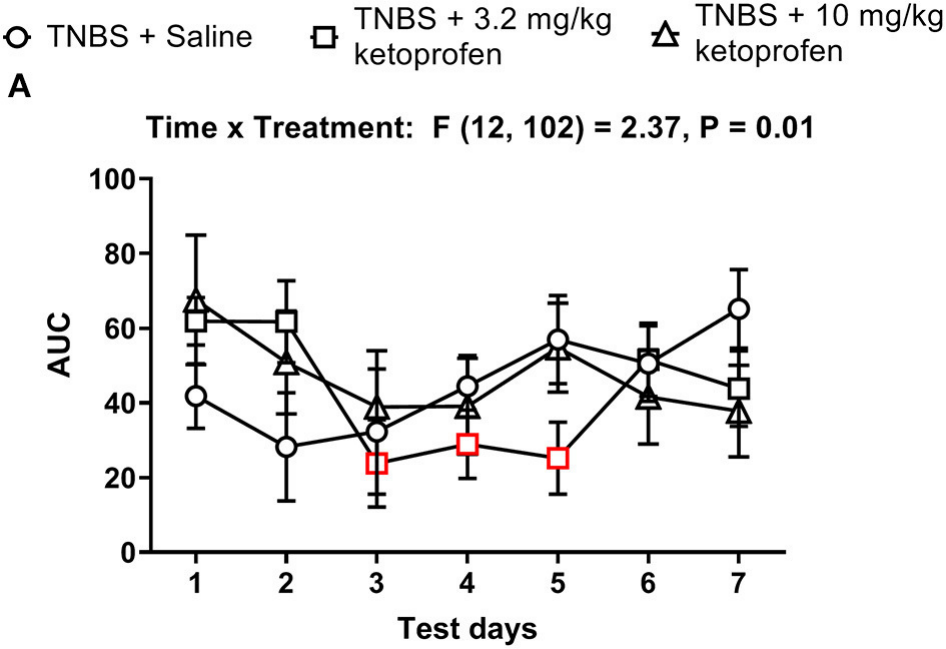
Time dependent effect of Ketoprofen on TNBS-induced nesting. **(A)** The area under the curve (AUC) of nesting scores recorded daily in mice treated with TNBS and saline, 3.2 or 10 mg/kg/day ketoprofen s.c. Red points indicate significance (*P* < 0.05) vs. day one by repeated measures two-way ANOVA with Dunnett's test.

Figures 13, 14 show the effect of daily administration of 3.2 or 10 mg/kg ketoprofen on TNBS-induced depression of nesting. Note that effects of ketoprofen alone were not tested here because previous studies have found no effect of these ketoprofen doses on nesting in mice (5–7). Analysis of the time course of nesting on each day indicated that unlike morphine neither dose of ketoprofen altered TNBS-induced nesting depression on any day (Figure 13). Thus, 2-way ANOVA did not detect a significant main effect of ketoprofen treatment on any day (Days 1, 2, 3, 4, 5, 6, and 7).

The AUC of nesting in TNBS-treated mice injected daily with ketoprofen (3.2 or 10 mg/kg) indicated that ketoprofen at any dose did not significantly affect TNBS-induced depression of nesting across 7 days (Figure 14). Thus, 2-way ANOVA of the AUC of nesting in mice treated with saline or ketoprofen on each day indicated a significant time x treatment interaction [F (12, 102) = 2.4, *P* = 0.01] and a significant main effect of time [F (6, 102) = 2.4, *P* = 0.03]; however, consistent with our analysis for Figure 13 we did not observe a main treatment effect of ketoprofen on nesting [F (2, 17) = 0.1, *P* = 0.92]. Altogether, the findings indicate that ketoprofen does not alter the intensity of the depression of nesting caused by TNBS.

**Figure 15:**
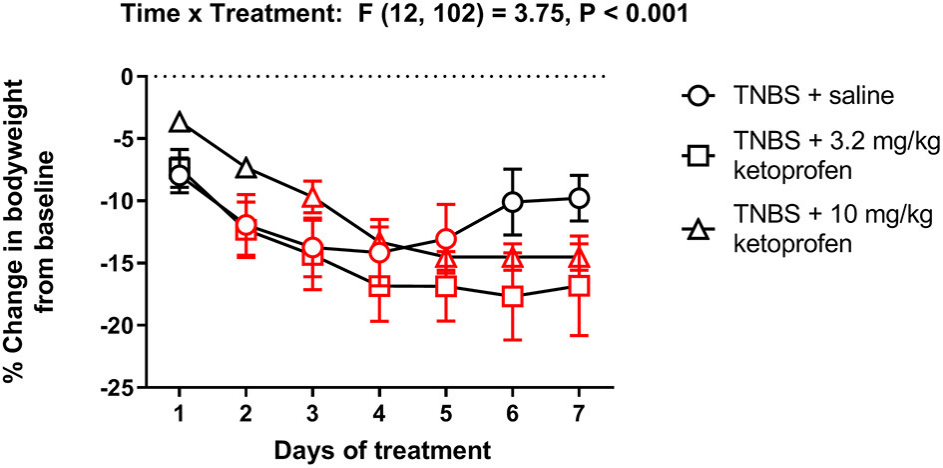
Effect of repeated ketoprofen administration on the body weight of TNBS-treated mice. Percent change in body weight from baseline of TNBS-treated mice injected with saline, 3.2 or 10 mg/kg/day ketoprofen s.c. Compared to saline, ketoprofen did not significantly alter the body weight of TNBS-treated mice. Data are mean ± SEM and analyzed by two-way repeated measures ANOVA. TNBS + saline (*N* = 8/day); TNBS + 3.2 mg/kg ketoprofen (*N* = 7/day); TNBS + 10 mg/kg ketoprofen (*N* = 5/day). Red points indicate significance (*P* < 0.05) vs. day one by repeated measures two-way ANOVA with Dunnett's test.

Figure 15 shows body weight changes in TNBS-treated mice repeatedly injected with saline, 3.2 or 10 mg/kg/day ketoprofen. Ketoprofen did not mitigate the TNBS-induced decrease in body weight [Treatment effect: F (2, 17) = 0.7, *P* = 0.49]. In fact, compared to the saline group mice repeatedly treated with ketoprofen did not appear to recover the lost body weight as indicated by the significant decrease in body weight on Day 7 from Day 1 in the TNBS + ketoprofen groups but not in the TNBS + saline controls.

## Discussion

In the present study, administration of TNBS significantly decreased nestlet consolidation in adult male mice over 7 days. Repeated treatment with morphine alone also transiently depressed nesting when delivered by repeated daily injections or by a morphine pellet. However, the combination of morphine and TNBS resulted in significant depression of nesting that did not recover over the 7 days at the 3.2 mg/kg morphine dose. We have previously shown that single TNBS administration results in significant inflammation as noted by histology of the colon, increase in the pro-inflammatory cytokine IL-1β and loss in body weight within 3 days post-administration (22). Furthermore, neither dose of ketoprofen alleviated the disruption of nesting caused by colonic inflammation after repeated administration. These findings suggest that, in mice, MOR agonist analgesics worsen the depression of nesting caused by colonic inflammation and provide support to clinical evidence in humans that opioids can reduce the quality of life of IBD patients (17, 23–25). The overall effects of TNBS and the combination of TNBS with morphine on nesting behavior correlated well with the locomotor assay. Colonic inflammation-induced depression of nesting behavior tended to recover over time as did effects on locomotor activity. Morphine alone at 3.2 mg/kg did not alter locomotion but interestingly depressed nesting on days 1–4. Importantly, the combination of TNBS and morphine markedly reduced nesting behavior and locomotion over the 7 days of testing. These findings suggest that ethologically relevant assays of pain-depressed behavior can be successfully utilized to evaluate the disruptive effects of IBD-induced inflammation and associated pain on activities of daily life and to assess the effectiveness of analgesics to reverse the depression of these behaviors.

Several reports have recently suggested that opioid analgesics might worsen the pathophysiology of IBD and significantly reduce the quality of life of IBD patients (17, 23–25). Accordingly, the aim of the present study was to investigate the effect of the MOR agonist analgesic morphine on nesting behavior of mice suffering from acute colitis induced by TNBS. Mice are highly motivated to build nests as a means of protection against predators and unfavorable environmental conditions (1, 26). They instinctively exhibit this behavior even in a laboratory setting. There is growing evidence that nest building and locomotor activity are susceptible to disruption by pain or distress and can provide an indication of animal well-being (4–6, 26–28). Therefore, in the present study we used nest building and locomotor activity as correlates of mouse welfare and assessed how induction of colitis by TNBS and treatment with the analgesic, morphine, altered animal behavior. Our findings suggest that opioids worsen the overall well-being of mice with colitis and provide evidence in support of clinical reports in humans that opioids reduce the quality of life of IBD patients. Moreover, this exacerbation of TNBS-induced depression of nesting and locomotion by morphine contrasts with the alleviation of TNBS-induced tactile hypersensitivity by morphine in mice (21) and suggests that drug effects on IBD related behavioral depression may translate more accurately to clinical findings in humans than drug effects on stimulated behaviors.

Inflammatory Bowel Disease (IBD) is characterized by inflammation that alters the architecture of the gut wall and is accompanied by abdominal pain (29). Previously, studies have reported increased latency to nest and reduced locomotor activity in a mouse dextran sulphate sodium (DSS)-colitis model (4, 30, 31). Consistent with these findings, we too initially observed significantly reduced nest building activity in mice treated acutely with TNBS. This was accompanied with a significant loss of body weight, a cardinal sign of colonic inflammation, and decreased locomotor activity. After 7 days, both nesting and locomotor activity tended to recover toward control values, however body weight remained below the vehicle controls. The cytokine response in the acute TNBS model has been reported to display a Th1/Th17 profile, consistent with Crohn's disease— related immune response (32). Further studies will be needed to evaluate the nesting behavior in chronic TNBS models which demonstrate an enhanced Th1/Th17 profile.

We have previously shown that TNBS-induced colonic inflammation mitigates the antinociceptive effects of morphine as tolerance develops at a faster rate in the presence of colonic inflammation (22). It is therefore likely that colonic inflammation also exacerbates morphine-induced depression of nesting and locomotor activity with repeated administration as a result of tolerance. We and others have previously shown that chronic morphine administration alters the gut microbiome, disrupts the epithelial barrier and enhances colonic inflammation as a result of bacterial translocation from the gut lumen (23, 33, 34). In the TNBS-acute colitis model, the gut wall is significantly damaged and the epithelial barrier is disrupted (22, 35). Since 3.2 mg/kg morphine alone did not disrupt locomotor activity, the exacerbated depression of nesting and locomotor activity, and failure to recover this intrinsic behavior could be due to an opioid-induced increase in the extent of colonic inflammation. Indeed, recently it was reported in a DSS-colitis mouse model that hydromorphone enhanced gut permeability, bacterial translocation, and intestinal inflammation (25). It is important to note that 3.2 mg/kg morphine alleviated TNBS-induced depression of locomotion on Day 1 but not nesting. This could be due to the acute antinociceptive effect of morphine on TNBS-induced depression of locomotion.

NSAIDs have been implicated as a risk factor in Crohn's disease in humans and are believed to worsen disease outcomes including relapse (19, 36, 37). However, other cross-sectional and cohort studies have not found an effect of NSAIDS on IBD (20, 38, 39). In the present study, ketoprofen at 3.2 and 10 mg/kg did not alleviate TNBS-induced nesting depression. Moreover, ketoprofen appeared to perturb the recovery of body weight in TNBS-treated mice. Ketoprofen and other NSAIDS can produce gastric ulceration that is dose dependent and cumulative with repeated treatment (40) and these gastrointestinal effects may limit the effectiveness of these drugs to alleviate IBD pain-related behavioral depression.

In summary, we demonstrate that TNBS depresses nesting behavior and locomotor activity. Although morphine alone did not induce nesting depression or alter locomotor activity over time, when administered to the TNBS-treated mice, morphine persistently depressed the nesting behavior and locomotor activity. Finally, the NSAID, ketoprofen, does not produce an effect on TNBS-induced inhibition of nesting. Consequently, the findings presented in this study suggest that opioids can significantly worsen the behavioral aspects of colonic inflammation. The mechanism for this may involve an exaggerated inflammation due to opioid-mediated bowel dysfunction including disruption of the gut epithelial barrier and reduced gastrointestinal motility. Our findings also suggest that assessing intrinsic behaviors such as nest building and locomotor activity is a sensitive tool to evaluate the clinical usefulness of analgesics in mitigating symptoms of inflammatory bowel diseases.

## Data Availability Statement

The raw data supporting the conclusions of this article will be made available by the authors, without undue reservation.

## Ethics Statement

The animal study was reviewed and approved by VCU IACUC.

## Author Contributions

SC, EKos, JJ, and EKom: conducted experiments and analyzed data. SC, KM, SS, and HA: conceptualized and prepared manuscript. All authors contributed to the article and approved submitted versions.

## Funding

This study was supported by National Institutes of Health grants: R25GM090084 (IMSD), R25GM089614 (PREP), P30DA033934, and R01DA036975. SC and EKom self-identify as an under-represented minority in science and were supported by the Center for Health Disparities at Virginia Commonwealth University. EKos was supported by TUBITAK (The Scientific and Technological Research Council of Turkey)/2214-A (International Doctoral Research Fellowship Programme) and YÖK (Council of Higher Education) 100/200 Doctoral Research Fellowship Programme.

## Conflict of Interest

The authors declare that the research was conducted in the absence of any commercial or financial relationships that could be construed as a potential conflict of interest.

## Publisher's Note

All claims expressed in this article are solely those of the authors and do not necessarily represent those of their affiliated organizations, or those of the publisher, the editors and the reviewers. Any product that may be evaluated in this article, or claim that may be made by its manufacturer, is not guaranteed or endorsed by the publisher.
